# Investigating the impact of open label design on patient‐reported outcome results in prostate cancer randomized controlled trials

**DOI:** 10.1002/cam4.3335

**Published:** 2020-08-26

**Authors:** Guillaume Mouillet, Fabio Efficace, Antoine Thiery‐Vuillemin, Emilie Charton, Mieke Van Hemelrijck, Francesco Sparano, Amélie Anota

**Affiliations:** ^1^ Department of Medical Oncology University Hospital of Besançon Besançon France; ^2^ Methodological and Quality of Life Unit in Oncology University Hospital of Besançon Besançon France; ^3^ INSERM EFS BFC UMR1098, Interactions Hôte‐Greffon‐Tumeur/Ingénierie Cellulaire et Génique University Bourgogne Franche‐Comté Besançon France; ^4^ Data Center and Health Outcomes Research Unit, Italian Group for Adult Hematologic Diseases (GIMEMA) Rome Italy; ^5^ Translational Oncology and Urology Research (TOUR) School of Cancer and Pharmaceutical Sciences King's College London Guy's Hospital London UK; ^6^ French National Platform Quality of Life and Cancer Besançon France

**Keywords:** blinded, health‐related quality of life, methodology, patient‐reported outcome, prostate cancer, randomized trials

## Abstract

**Background:**

While open‐label randomized controlled trials (RCT) are common in oncology, some concerns have been expressed with regard to Patient‐Reported Outcomes (PRO)‐based claims stemming from these studies. We aimed to investigate the impact of open‐label design in the context of prostate cancer (PCa) RCTs with PRO data.

**Methods:**

Randomized controlled trials of PCa with a PRO endpoint published between 2004 and 2018 were considered. RCTs were systematically evaluated on the basis of previously defined criteria, including international PRO reporting quality standards and the Cochrane Collaboration's tool for assessing Risk of Bias. The rate of concordance was estimated and compared between traditional clinical outcomes (eg, survival or tumor response) and PRO in open and blinded RCTs.

**Results:**

We identified 110 RCTs published between 2004 and 2018, of which 62% (n = 68) were open‐label. The general characteristics of PCa RCTs were not different according to their design (open‐label vs blinded). The proportion of PCa RCTs with high‐quality PRO reporting was not different between open‐label RCTs and blinded RCTs (41.2% vs 38.1%; *P* = .75). No statistically significant difference was found between PRO results and concordance with traditional clinical outcomes according to the study design.

**Conclusion:**

Our findings suggest that there is no evidence of significant bias for PROs due to the absence of blinding in the context of PCa RCTs. Further analyses should be conducted in other cancer disease sites.

## Graphical abstract

The systematic review of phase III in prostate cancer suggests that there is no evidence of significant bias on PRO results due to the absence of blinding.

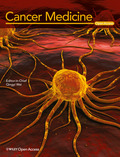

## 1 INTRODUCTION

Being the most common cancer in males,^1^ it is not surprising that prostate cancer (PCa) has a severe impact on the burden of disease. Its various treatments (eg, radical prostatectomy, androgen deprivation therapy, chemotherapy) come with a number of potential side effects^2^, ^3^, ^4^ and hence have an effect on health‐related quality of life (HRQoL).^5^, ^6^, ^7^ The latter is therefore also an important factor when treatment choices have to be made.^8^, ^9^, ^10^, ^11^ Both the US Food and Drug Administration (FDA) and the European Medicines Agency (EMA) highly endorse the use of patient‐reported outcomes (PROs) in this context by requiring the integration of the patients’ perspective through better reporting of adverse events and HRQoL in randomized controlled trials (RCTs).^12^, ^13^

However, several systematic reviews have highlighted that a high proportion of RCTs including PROs poorly report on these measurements, with missing information being very common.^14^, ^15^, ^16^ Another important methodological issue with the reporting of PROs in RCTs is the open‐label setting.^17^, ^18^ Hence, the FDA rarely considers open‐label RCTs adequate for PRO based claims.^19^, ^20^, ^21^ Nonblinded patients may report symptoms and adverse events differently compared to blinded patients.^22^ Moreover, open‐labeling may result in patients assigned to the control group being more likely to drop out, while patients in the experimental group being more likely to complete their PRO monitoring.^23^, ^24^, ^25^ Some concerns with respect to PRO reporting have also been expressed for RCTs with unintentional unblinding when treatments have specific toxicities.^22^, ^26^

Nevertheless, open‐labeling is common in oncology RCTs due to practical restrictions,^20^, ^27^ hence it may be a challenge to integrate PRO measurement in oncology clinical trials and meet regulators' requirements.^12^, ^28^ To the best of our knowledge, there is no published data systematically investigating the impact of open‐labeling in the context of PCa RCTs with PRO data. We therefore aimed to compare the proportion of concordance and discordance between traditional clinical outcomes and PROs in open‐label and blinded PCa RCTs.

## 2 METHODS

### 2.1 Data selection

The analysis reported here was based on data collected from the large Patient‐Reported Outcome Measurements Over Time In ONcology (PROMOTION) database.^29^ This registry (promotion.gimema.it) includes all cancer RCTs that have included at least one PRO, either as a primary or secondary/exploratory study endpoint, published since 2004 identified through systematic literature searches in electronic databases (eg, PubMed/MEDLINE). The registry intends to facilitate the evaluation of the quality of RCT‐based PRO assessment methodology, instruments, statistical analysis and reporting.^29^ For this analysis, all RCTs of PCa published between January 2004 and June 2018 were considered.

Details of inclusion criteria and methodology to evaluate studies have been described previously.^30^ Briefly, all RCTs comparing different conventional medical treatment modalities and symptom management enrolling at least 50 patients with PCa (combined arms) were studied. Studies assessing prevention or screening programs, complementary or alternative medicine or psychosocial intervention were excluded. The search was restricted to English language articles. If a selected study had multiple publications, we incorporated all relevant papers in the analysis. More specifically for this update, four reviewers independently reviewed all identified studies, and a fifth reviewer was consulted in case of disagreement.

We specifically collected information on “blinding of participant” using the Cochrane Risk of Bias tool. The Cochrane Risk of Bias tool provides a framework for assessing risk of bias in studies included in a systematic review.^31^ The tool covers six domains of bias: selection bias (random sequence generation; allocation concealment), performance bias (blinding of participants and personnel), detection bias (blinding outcome assessment), attrition bias (incomplete outcome data), reporting bias (selective reporting), and other possible bias. Performance bias is focused on blinding of participants and personnel and quantified as ‘low’, ‘high’ or ‘unclear’. This review classified the RCTs into two groups: (a) “open‐label trial” because of a high risk of performance bias and (b) “blinded trial” because of a low risk of performance bias. RCTs with performance bias classified as “unclear” were reviewed again by two of the reviewers to reclassify as “open‐label” or “blinded” (GM and AA).

### 2.2 Concordance between PRO and traditional endpoint results

For each trial we calculated concordance between PROs and more traditional clinical outcomes. For the purpose of this review, we will refer to “clinical outcomes” to identify any type of non‐PRO assessment (eg, such as survival outcomes, adverse events or tumor response), used as endpoints in each considered RCT. Each PRO was assessed as “better”, “no difference”, or “worse” compared to the experimental to control arms. For example, if more than half of the PRO dimensions that were statistically significant were in favor of the experimental arm, the PRO results were considered as “better”. If none of the PRO dimensions were statistically significant, or if half of the PRO dimensions were in favor of each treatment arm, the PRO results were classified as “no difference”. Trials reporting only descriptive results for PRO endpoints were thus excluded from this analysis. For the clinical outcomes, the same classification was then performed. We then calculated the rate of concordance for clinical outcomes and PRO in open and blinded RCTs.

In addition, we evaluated the quality of PRO reporting in open‐label vs. blinded RCTs according to International Society for Quality of Life Research (ISOQOL) PRO recommended criteria,^30^, ^32^ which laid the groundwork for the subsequent development of the CONSORT‐PRO extension.^33^ Studies were categorized as “high quality of PRO reporting” if at least 20 out of 29 (for primary endpoints) criteria were satisfied (or 12 out of 18 for secondary endpoints). Differences in reporting between open‐label and blinded RCTs were then quantified using the chi‐square test performed at the statistical level of 5%.

Qualitative variables are described as absolute and relative frequencies. Chi‐square test or Fisher's exact test was used to compare qualitative variables. All tests were two‐sided at the statistical level of 5%. All analyses were conducted on SAS software version 9.4 (SAS Institute Inc).

## 3 RESULTS

### 3.1 General results

**FIGURE 1:**
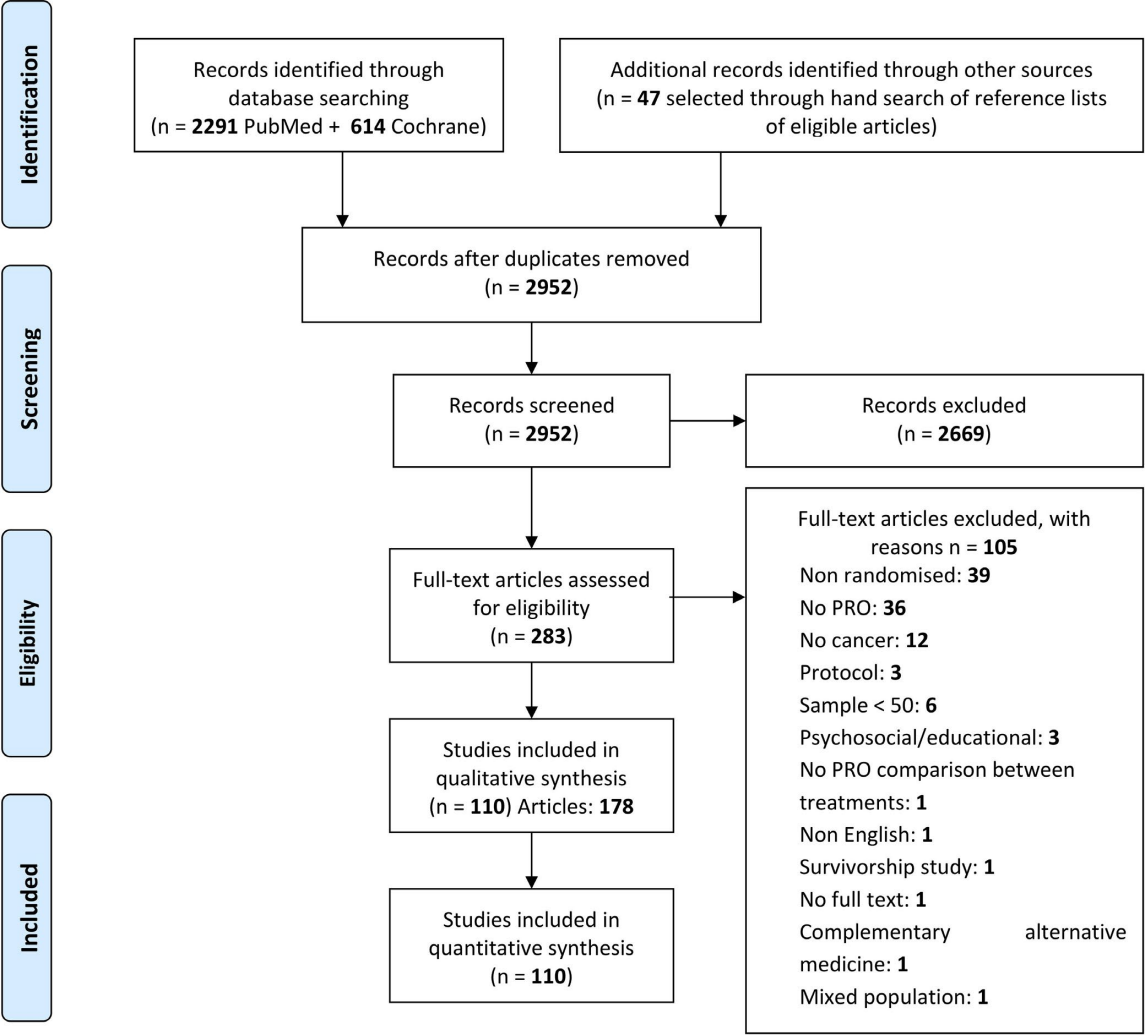
Schematic breakdown of literature search results of Prostate Randomized Controlled Trials (Preferred Reporting Items for Systematic Reviews and Meta‐analysis).

A total of 110 RCTs were identified according to our predefined selection criteria among 2,952 records screened between January 2004 and June 2018. Figure 1 shows the Flowchart for the inclusion and exclusion of PCa RCTs.

Among all the 110 RCTs analyzed, 68 (61.8%) were nonblinded/open‐label studies and 42 (38.2%) were blinded to the patients at least (Table 1). A total of 66 (60.0%) RCTs had an overall sample size > 200 patients, 45 (40.9%) were conducted in more than one country, and 65 (59.1%) were supported by industry.

**TABLE 1:** Randomized clinical trial (RCT) demographic characteristics according to blinding of participants and personnel

Variable	Total		Open‐Label		Blinded		
	No.	(%)	No.	(%)	No.	(%)	*P*
	110	(100)	68	(100)	42	(100)	
Basic RTC demographics							
International							.13
No	65	(59.1)	44	(64.7)	21	(50.0)	
Yes	45	(40.9)	24	(35.3)	21	(50.0)	
Industry supported (fully or in part)							<.001
No	45	(40.9)	37	(54.4)	8	(19.1)	
Yes	65	(59.1)	31	(45.6)	34	(80.9)	
Overall study sample size							.38
≤200 patients	44	(40.0)	25	(36.8)	19	(45.2)	
>200 patients	66	(60.0)	43	(63.2)	23	(54.8)	
Disease stage							.04
Only Advanced / metastatic	43	(39.1)	21	(30.9)	22	(52.4)	
Only non‐metastatic / local	47	(42.7)	31	(45.6)	16	(38.1)	
Both	20	(18.2)	16	(23.5)	4	(9.5)	
Broad treatment type							
Radiotherapy	36	(32.7)	25	(36.8)	11	(26.2)	.25
Surgery	14	(12.7)	9	(13.2)	5	(11.9)	.84
Chemotherapy	21	(19.1)	12	(17.7)	9	(21.4)	.62
HT	45	(40.9)	31	(45.6)	14	(33.3)	.20
Difference between treatment arms in the clinical primary end point							.87
No	40	(44.0)	26	(43.3)	14	(45.2)	
Yes	51	(56.0)	34	(56.7)	17	(54.8)	
OS difference favouring experimental treatment							.66
No	42	(38.2)	28	(41.2)	14	(33.3)	
Yes	13	(11.8)	7	(10.3)	6	(14.3)	
N/A (in case OS was not assessed)	55	(50.0)	33	(48.5)	22	(52.4)	
PRO‐related basic characteristics							
Most frequent PRO instruments							
EORTC questionnaires	31	(28.2)	22	(32.4)	9	(21.4)	.22
FACT questionnaires	27	(24.6)	15	(22.1)	12	(28.6)	.44
Visual Analogue Scale	11	(10.0)	6	(8.8)	5	(11.9)	.60
Length of PRO assessment during RCT							.05
≤6 mo	29	(26.4)	17	(25.0)	12	(28.6)	
≤1 y	20	(18.2)	12	(17.7)	8	(19.0)	
>1 y	57	(51.8)	39	(57.3)	18	(42.9)	
Unknown	4	(3.6)	0	(0)	4	(9.5)	
Secondary paper on PRO							.94
No	79	(71.8)	49	(72.1)	30	(71.4)	
Yes	31	(28.2)	19	(27.9)	12	(28.6)	

Abbreviations: EORTC, European Organization for Research and Treatment of Cancer; FACT, Functional Assessment of Cancer Therapy; HT, hormone therapy; OS, overall survival; PRO, patient‐reported outcomes; RCT, randomized controlled trial.

A large part of the RCTs included patients with locoregional PCa (42.7%, n = 47), and hormonal treatment was most frequently used (40.9%, n = 45). A statistically significant difference between treatment arms in the clinical primary endpoint was found in 51 (56.0%) RCTs.

With respect to the PRO components, 38 RCTs (34.6%) had a PRO measure as primary endpoint. PRO results were detailed in a secondary paper for 31 RCTs (28.2%). The general characteristics of PCa RCTs were not statistically different according to their design (open‐label vs blinded) except the disease stage and that a majority of blinded RCTs were industry supported (80.9%, n = 34; *P *< .001).

### 3.2 Impact of blinding on PRO results

Analysis of concordance with clinical outcomes was conducted on 98 RCTs (37 blinded RCTs and 61 open‐label RCTs), since 12 studies only reporting descriptive PRO results were excluded. The proportion of RCTs reporting a difference between treatment arms in the primary endpoint was not different between blinded and open‐label RCTs.

Among the 55 RCTs reporting better clinical outcomes in favour of the experimental arm, 56.4% (n = 31) reported better PRO, 25.4% (n = 14) reported PRO equivalence and 18.2% (n = 10) reported worse PRO in the experimental arm. Of the 36 RCTs reporting clinical outcomes not different between arms, PROs were reported to be better in the experimental arm in 36.1% (n = 13) of the RCTs, were not different in 55.6% (n = 20), and worse in 8.3% (n = 3).

More specifically, for the blinded RCTs which reported better clinical outcomes favoring the experimental arm (n = 19), 84.2% (n = 16) reported better PRO favoring the experimental arm, 10.5% (n = 2) reported equivalent PRO and 5.3% (n = 1) reported worse PRO for the experimental arm. For the open‐label RCTs these proportions were 41.7% (n = 15), 33.3% (n = 12), and 25.0% (n = 9), respectively.

Finally, no statistically significant difference was found between PRO results and concordance with clinical outcomes according to the status of the study (ie, blinded or not to the patients) (Table 2). For the RCTs reporting equivalent clinical outcome or nondifference between arms, better PROs were reported in 35.0% (n = 7) of the open‐label trials and 37.5% (n = 6) of the blinded RCTs. The proportions of RCTs which reported no difference in PRO among those reporting no difference in clinical outcomes were also consistent across subgroups, with 55.6% of all RCTs, 55% of open‐label, and 56.2% of blinded RCTs.

**TABLE 2:** Influence of blinding of participants on patient‐reported outcome results

Type of trial	All cohort (n = 98)				Open‐label (N = 61)				Blinded (N = 37)			
PRO difference between treatment arms	Better PRO N (%)	No difference N (%)	Worse PRO N (%)	Total N	Better PRO N (%)	No difference N (%)	Worse PRO N (%)	Total N	Better PRO N (%)	No difference N (%)	Worse PRO N (%)	Total N
Traditional clinical endpoint difference between arms												
Better Clinical Outcomes	31 (56.4)	14 (25.4)	10 (18.2)	55	15 (41.7)	12 (33.3)	9 (25.0)	36	16 (84.2)	2 (10.5)	1 (5.3)	19
No difference	13 (36.1)	20 (55.6)	3 (8.3)	36	7 (35.0)	11 (55.0)	2 (10.0)	20	6 (37.5)	9 (56.2)	1 (6.3)	16
Worse Clinical Outcomes	3 (42.8)	2 (28.6)	2 (28.6)	7	2 (40.0)	1 (20.0)	2 (40.0)	5	1 (50.0)	1 (50.0)	0 (0.00)	2
Total	47	36	15	98	24	24	13	61	23	12	2	37

Note

Results are classified as better PRO if they are favoring the experimental arm, equivalence if no difference at all is observed, or half of the dimensions are favoring each treatment arm, and worse PRO if they are favoring the standard arm. The same classification was made for clinical endpoints.

All chi‐square tests were nonsignificant.

### 3.3 Quality of PRO reporting by RCT designs

The quality of reporting was globally equivalent between open‐label and blinded RCTs (Table 3). However, the rationale for the choice of the PRO instrument was more frequently provided in open‐label RCTs (66.2% vs 42.9%, *P* = .02). Conversely, additional details regarding the hypothesis of PRO analysis and post hoc analyses were found in a higher proportion of blinded RCTs (8.7% vs 33.3% *P* = .09 and 27.9% vs 57.1%, *P *< .01 respectively).

**TABLE 3:** Patient‐reported outcomes (PRO) reporting according to ISOQOL PRO Guidelines and study design

Variable	Total		Open‐label		Blinded		*P*
	No.	(%)	No.	(%)	No.	(%)	
	110	(100)	68	(100)	42	(100)	
**Title and abstract**							
The PRO should be identified as an outcome in the abstract.							.69
No	9	(8.2)	5	(7.3)	4	(9.5)	
Yes	101	(91.8)	63	(92.7)	38	(90.5)	
*The title of the paper should be explicit as to the RCT including a PRO* *							.63
No	17	(44.7)	11	(47.8)	6	(40.0)	
Yes	21	(55.3)	12	(52.2)	9	(60.0)	
**Introduction, background, and objectives**							
The PRO hypothesis should be stated and specify the relevant PRO domain, if applicable.							.58
No	26	(23.6)	16	(23.5)	10	(23.8)	
Yes	36	(32.7)	20	(29.4)	16	(38.1)	
N/A (if explorative)	48	(43.7)	32	(47.1)	16	(38.1)	
*The introduction should contain a summary of PRO research that is relevant to the RCT* *							.22
No	9	(23.7)	7	(30.4)	2	(13.3)	
Yes	29	(76.3)	16	(69.6)	13	(86.7)	
*Additional details regarding the hypothesis should be provided, including the rationale for the selected domains, the expected directions of change, and the time points for assessment* *							0.09
No	31	(81.6)	21	(91.3)	10	(66.7)	
Yes	7	(18.4)	2	(8.7)	5	(33.3)	
**Methods**							
***Outcomes***							
The mode of administration of the PRO tool and the methods of collecting data should be described							.61
No	89	(80.9)	54	(79.4)	35	(83.3)	
Yes	21	(19.1)	14	(20.6)	7	(16.7)	
The rationale for the choice of the PRO instrument used should be provided.							.02
No	47	(42.7)	23	(33.8)	24	(57.1)	
Yes	63	(57.3)	45	(66.2)	18	(42.9)	
Evidence of PRO instrument validity and reliability should be provided or cited.							.23
No	31	(28.2)	17	(25.0)	14	(33.3)	
Yes, for All PRO instruments	54	(49.1)	32	(47.1)	22	(52.4)	
Yes, only for some PRO instruments	25	(22.7)	19	(27.9)	6	(14.3)	
The intended PRO data collection schedule should be provided.							.60
No	11	(10.0)	6	(8.8)	5	(11.9)	
Yes	99	(90.0)	62	(91.2)	37	(88.1)	
PRO should be identified in the trial protocol; post hoc analyses should be identified.							<.01
No	67	(60.9)	49	(72.1)	18	(42.9)	
Yes	43	(39.1)	19	(27.9)	24	(57.1)	
The status of PRO as either a primary or secondary outcome should be stated.							.07
No	11	(10.0)	10	(14.7)	1	(2.4)	
Yes	88	(80.0)	50	(73.5)	38	(90.5)	
Unclear	11	(10.0)	8	(11.8)	3	(7.1)	
*A citation for the original development of the PRO instrument should be provided* *							.57
No	14	(36.8)	7	(30.4)	7	(46.8)	
Yes	13	(34.2)	9	(39.2)	4	(26.7)	
Yes, only for some PRO instruments	11	(29.0)	7	(30.4)	4	(26.7)	
*Windows for valid PRO responses should be specified and justified as being appropriate for the clinical context* *							.46
No	18	(47.4)	12	(52.2)	6	(40.0)	
Yes	20	(52.6)	11	(47.8)	9	(60.0)	
***Sample size***							
*There should be a power sample size calculation relevant to the PRO based on a clinical rationale* *							.72
No	14	(36.8)	9	(39.1)	5	(33.3)	
Yes	24	(63.2)	14	(60.9)	10	(66.7)	
***Statistical methods***							
There should be evidence of appropriate statistical analysis and tests of statistical significance for each PRO hypothesis tested.							.57
No	5	(4.5)	3	(4.4)	2	(4.8)	
Yes	33	(30.0)	18	(26.5)	15	(35.7)	
N/A (if PRO hypotheses were not stated)	72	(65.5)	47	(69.1)	25	(59.5)	
The extent of missing data should be stated.^a^							.44
No	32	(29.1)	18	(26.5)	14	(33.3)	
Yes	78	(70.9)	50	(73.5)	28	(66.7)	
Statistical approaches for dealing with missing data should be explicitly stated.^a^							.68
No	81	(73.6)	51	(75.0)	30	(71.4)	
Yes	29	(26.4)	17	(25.0)	12	(28.6)	
*The manner in which multiple comparisons have been addressed should be provided* *							.80
No	27	(71.1)	16	(69.6)	11	(73.3)	
Yes	11	(28.9)	7	(30.4)	4	(26.7)	
**Results**							
***Participant flow***							
A flow diagram or a description of the allocation of participants and those lost to follow‐up should be provided for PRO specifically.							.37
No	61	(55.5)	40	(58.8)	21	(50.0)	
Yes	49	(44.5)	28	(41.2)	21	(50.0)	
The reasons for missing data should be explained.							0.60
No	70	(63.6)	42	(61.8)	28	(66.7)	
Yes	40	(36.4)	26	(38.2)	14	(33.3)	
***Baseline data***							
The study patients’ characteristics should be described, including baseline PRO scores.							.88
No	35	(31.8)	22	(32.4)	13	(30.9)	
Yes	75	(68.2)	46	(67.6)	29	(69.1)	
***Outcomes and estimation***							
Are PRO outcomes also reported in a graphical format?^b^							.44
No	39	(35.5)	26	(38.2)	13	(30.9)	
Yes	71	(64.5)	42	(61.8)	29	(69.1)	
*The analysis of PRO data should account for survival differences between treatment groups, if relevant* *							.45
No	1	(2.6)	1	(4.3)	0	(0)	
Yes	5	(13.2)	2	(8.7)	3	(20.0)	
N/A (if not relevant)	32	(84.2)	20	(87.0)	12	(80.0)	
*Results should be reported for all PRO domains (if multidimensional) and items identified by the reference instrument* *							.97
No	10	(26.3)	6	(26.1)	4	(26.7)	
Yes	28	(73.7)	17	(73.9)	11	(73.3)	
*The proportion of patients achieving predefined responder definitions should be provided where relevant* *							.11
No	4	(10.5)	1	(4.4)	3	(20.0)	
Yes	7	(18.4)	3	(13.0)	4	(26.7)	
N/A (if not relevant)	27	(71.1)	19	(82.6)	8	(53.3)	
**Discussion**							
***Limitations***							
The limitations of the PRO components of the trial should be explicitly discussed							.60
No	70	(63.6)	42	(61.8)	28	(66.7)	
Yes	40	(36.4)	26	(38.2)	14	(33.3)	
***Generalizability***							
Generalizability issues uniquely related to the PRO results should be discussed.							.42
No	47	(42.7)	27	(39.7)	20	(47.6)	
Yes	63	(57.3)	41	(60.3)	22	(52.4)	
***Interpretation***							
Are PRO interpreted (not just restated)?^b^							.23
No	28	(25.5)	20	(29.4)	8	(19.1)	
Yes	82	(74.5)	48	(70.6)	34	(80.9)	
The clinical significance of the PRO findings should be discussed.							.12
No	74	(67.3)	42	(61.8)	32	(76.2)	
Yes	36	(32.7)	26	(38.2)	10	(23.8)	
The PRO results should be discussed in the context of the other clinical trial outcomes.							.19
No	15	(13.6)	7	(10.3)	8	(19.1)	
Yes	95	(86.4)	61	(89.7)	34	(80.9)	
**Other information**							
***Protocol***							
*A copy of the instrument should be included if it has not been published previously* *							.35
No	15	(39.5)	11	(47.8)	4	(26.7)	
Yes	12	(31.5)	7	(30.4)	5	(33.3)	
N/A (if the instrument is already published or known in the literature)	11	(29.0)	5	(21.8)	6	(40.0)	

^a^ These items were originally combined in the ISOQOL recommended standards but have been split in this report to better investigate possible discrepancies between documentation of PRO missing data (ie, reporting how many patients did not complete a given questionnaire at any given time point) versus actual reporting of statistical methods to address this issue.

^b^ These items were not included in the ISOQOL recommended standards but have been evaluated in our study and reported in this table to have a wider outlook on the level of reporting.

* Additional standards only for PRO as primary outcome, number of trials = 38.

The status of PRO as either a primary or secondary endpoint was stated more frequently in blinded RCTs, albeit this difference was not statistically significant (73.5% vs 90.5%, *P* = .07). The extent of missing data was stated in 73.5% and 66.7% of the open‐label and blinded trials respectively, while the statistical approaches for dealing with these are less frequently reported (25% and 28.6%). Overall, the proportion of PCa RCTs with high‐quality reporting was not different between open‐label RCT and blinded RCT (41.2% vs 38.1%; *P* = .75) (Table 4).

**TABLE 4:** Prevalence of high quality of patient‐reported outcome reporting according to study design

	Total		Open‐label		Blinded		
	No.	(%)	No.	(%)	No.	(%)	*P*
	110	(100)	68	(100)	42	(100)	
Quality PRO reporting							.75
High	44	40.0	28	41.2	16	38.1	
Low	66	60.0	40	58.8	26	61.9	

Note

We defined an RCT as being of high quality, regarding the PRO assessment, if at least 12 of 18 (for PROs as secondary endpoint) or 20 of 29 (primary endpoint) of the ISOQOL recommended criteria were met.

## 4 DISCUSSION

When comparing concordance between traditional clinical outcomes and PROs between open‐label and blinded RCTs for PCa, we identified 110 RCTs published between 2004 and 2018. The majority of published trials were open‐label (62%) and concordance between PRO and clinical outcomes was not different between the two types of RCT study design.

In oncology clinical research, PROs complement other clinical outcomes such as survival, and adverse events assessed by the physicians and allow to incorporate the patient experience in the development of new drugs. A recent review of PRO labeling for oncology drugs approved by the FDA, and the EMA highlighted that among 49 oncology drugs approved between 2012 and 2016, no FDA PRO labeling was identified. While various reasons were noted, a key reason was also related to the open‐label design of RCTs.^21^

Bias may occur in open‐label trials, as observer bias and disappointment bias.^34^, ^35^, ^36^, ^37^ Therefore, according to the FDA, patients may be prone to provide biased reports of their own symptoms if they are aware of the treatment they received and lead to an overestimation of the treatment difference observed between the two treatment arms. Disappointment bias may affect dropout, and missing data when patients are assigned to the control group.^23^ In two recent publications, Roydhouse and colleagues have explored PRO completion rates between study arms in randomized open‐label and double‐blind cancer trials submitted to the FDA.^20^, ^38^ Their work underlined that differences favoring the experimental arm were seen only in four RCTs in which substantial between‐arm completion rate differences were observed. However, completion rates were high, and comparable between arms in a majority of open‐label RCTs.^20^, ^38^

Because open‐label designs are rather frequent in RCTs, some recommendations to help PRO results to impact labeling decisions in these (ie, open‐labels) research settings have been proposed: well‐designed RCT, well‐defined and adequate PRO measures, optimized PRO questionnaire completions rates, minimization of missing data, documentation of missing data, demonstration of large magnitude of effect, and possible consideration of follow‐up studies with PROs.^18^, ^39^

In our systematic review, the proportion of open‐label PCa RCTs is comparable to those generally observed in previous reviews.^18^, ^20^ We found that the results of the PROs were not consistently in favor of the experimental arm in open‐label RCTs. Another review by Atkinson and colleagues identified five double‐blind negative RCTs that reported no significant difference in PROs between study arms despite imbalances in multiple toxic effects.^40^ The authors concluded that these results might suggest that there is no sufficient bias to affect PRO between arms. Therefore, taken together with our findings, current evidence‐based data do not support previous concerns expressed with regard to the negative impact of open‐label design on overall quality of PRO findings.

There is a risk of a global devaluation of PRO relevance to systematically consider with suspicion PRO results in open‐label trials. Recent reviews pointed out that PRO reporting is far from the high‐quality standards emphasized by regulatory stakeholders and panel expert recommendations.^16^ Only 30% of the trials submitted by the sponsor to the FDA reported PRO compliance.^38^ Furthermore, in our analysis, the quality of PRO reporting according to ISOQOL recommendations was globally equivalent between open‐label and blinded RCTs. However, the overall quality of the reports is far from what we would expect as highlighted in a recent review.^16^

However, it is difficult to provide a definitive answer on the actual role of the open‐label design on PROs in RCT settings. To further explore it, a case‐control study or meta‐analysis which includes RCTs evaluating the same treatment in open‐label and blinded RCTs and using the same PRO questionnaires could provide additional insights. Recent large international initiatives have been set up to provide guidelines to help standardize the analysis of HRQoL and other PRO measures in cancer RCTs as well as help design PRO in trial protocols.^41^, ^42^, ^43^ These recommendations emphasize the need to reach high methodological quality in PRO researches.

Our study has limitations that should be noted. Our analysis was exploratory and may not be calibrated in terms of statistical power to detect a difference. Also, we could not get into details of the RCTs and explain for each RCT why PRO results were better or worse. Furthermore, RCTs included in the analysis were heterogenous in terms of therapies and setting (localized vs metastatic castration‐resistant PCa) which can have a different impact on PROs. Future works should focus on a specific disease state to confirm these results. Finally, the impact of open‐label design on compliance could not be assessed in our systematic review since we did not collect data about the rate of missing data.

This study also has strengths. To the best of our knowledge, it is the first evidence to systematically examine the risk of bias in open‐label RCTs in PCa, and analyses were based on a large number of studies published over the last several years. Also, our evaluation was based on internationally endorsed state of the art PRO reporting quality criteria.

To conclude, our findings suggest that there is no evidence of significant bias for PROs due to the absence of blinding in the context of PCa RCTs. Since the research question addressed in our work is not only relevant to PCa RCTs, further analyses should also be conducted to evaluate whether these results may extend to RCTs conducted in other cancer disease sites.

## CONFLICTS OF INTEREST

The authors have stated that they have no conflicts of interest.

## AUTHOR CONTRIBUTIONS

FE and FS designed and directed the database; GM, AA and FS collected the data related to this manuscript; GM and AA analyzed the data, and FS designed the figure; GM and AA wrote the manuscript with input from all authors. All authors provided critical feedback and helped shape the research, analysis and manuscript.

## Data Availability

Data available on request from the authors.
